# The First Sign of Consumption

**Published:** 1881-10

**Authors:** 


### THE FIRST SIGN OF CONSUMPTION.

It is not as extensively known as it ought to be, that, in the large majority of cases, consumption begins with *a slight cough in the morning on getting up*. After a while it is perceived at night on going to bed; next, there is an occasional "coughing spell" some time during the night; by this time there is a difficulty of breathing on any slightly unusual exercise, or in ascending a hill.

Even before this, persons begin to feel weak, while there is an almost imperceptible thinning in flesh, and a gradual diminution in weight—harassing cough, loose bowels, difficult breathing, swollen extremities, daily fever, and a miserable death! Miserable, because it is tedious, painful, and inevitable. How much it is to be wished that the symptoms of this hateful disease were more generally studied and understood, that it might be detected in its first insidious approaches, and application be made at once for its arrest and total eradication; for certain it is that, in very many instances, it could be accomplished.

It must be remembered, that cough is not an invariable attendant of consumption of the lungs, inasmuch as persons have died, and on examination, a large portion of the lungs were found to have decayed away, and yet these same persons were never noticed to have had a cough, or observed it themselves, until within a few days of death. But such instances are rare, and a habitual cough on getting up, and on going to bed, may be safely set down as indicating consumption begun. Cough as just stated, is originally a curative process, the means which nature uses to rid the body of that which offends, of that which is foreign to the system, and ought to be out of it; hence the folly of using medicines to keep down the cough, as all cough remedies sold in the shops merely do, without taking means at the same time for removing that state of things which makes cough necessary.

